# Acknowledgement to Reviewers of *Membranes* in 2014

**DOI:** 10.3390/membranes5010011

**Published:** 2015-01-08

**Authors:** Membranes Editorial Office

**Affiliations:** MDPI AG, Klybeckstrasse 64, CH-4057 Basel, Switzerland

The editors of *Membranes* would like to express their sincere gratitude to the following reviewers for assessing manuscripts in 2014:

Abbas, Zareen
Aili, David
Alcoutlabi, Mataz
Alpatova, Alla
Anglani, Franca
Ansón-Casaos, A.
Ayscough, Kathryn
Band, Hamid
Barbieri, Giuseppe
Baumgart, Tobias
Brin, Mitchell F.
Bringas, Eugenio
Büchs, Jochen
Buhler, K
Calles, José Antonio
Casado, Clara
Chang, Yung
Choquet, Daniel
Christensen, Erik Ilsø
Christie, MacDonald J.
Chung, Tai-Shung Neal
Clochard, Marie-Claude
Coronas, Joaquín
Cortese, Katia
de Graaff, Marthe
De Meyts, Pierre
Dedeoglu, Alpaslan
Diano, N.
Dimova, Rumiana
Drioli, Enrico
Ezeji, Thaddeus C.
Fatyeyeva, Kateryna
Fillaudeau, Luc
Fisher, Janet L.
Fullerton-Shirey, Susan K.
Garcia-Castello, E.M.
Ginsberg, Stephen D.
Gooley, Andrew
Gorri, Daniel
Gray, Stephen
Grilli, Selene
Gude, Veera Gnaneswar
Haystead, Timothy A. J.
Higuchi, Akon
Hoflack, Bernard
Hofs, Bas
Hu, Chien-Chieh
Hubbard, Stevan R.
Ito, Akira
Iwasaki, Yasuhiko
Ji, Peng
Jiménez-Barbero, Jesús
Jin, Xue
Joel, Dacks
Jones, Arwyn Tomos
Jönsson, A.-S.
Karabelas, A.J.
Khayet, Mohamed
Kim, Dae Eun
Kim, Tak-Hyun
Kimura, Katsuki
Koland, John G.
Kulozik, Ulrich
Kyu, Thein
Lai, Wei-Chi
Levy, Finn Olav
Li, Mingheng
Lin, Dar-Jong
Liu, Ying-Ling
Lloret, Lucia
Lo, Huang-Mu
Ludwig, Roland
Luis, Patricia
Maasten, Susan
Mancera, Ricardo L.
Maricq, Andres V.
Martin, Kelly J.
Martini, Claudia
Marullo, Stefano
Matsuyama, Hideto
Mayer, D.C. Ghislaine
Menkhaus, Todd J.
Moeller, Marcus J.
Möller, Heiko M.
Molnár, Elek
Nair, Sankar
Ng, How Yong
Nicoll, Roger A.
Nielsen, Per Halkjær
Nørgaard, C.F.
Omosebi, Ayokunle
Ortiz, Inmaculada
Owen, David
Raghavan, Malini
Rajendran, Lawrence
Rana, D.
Rhee, Jeong Seop
Rodrigues, Alírio E.
Rodriguez-Boulan, Enrique
Romanos, George E.
Saito, Hiromu
Sanciolo, Peter
Satyawali, Yamini
Scholich, Klaus
Schu, Peter
Sencadas, Vitor
Simpson, Fiona
Sioutopoulos, Dimitrios
Skirtach, André G.
Sotoft, Lene Fjerbaek
Staudt, Claudia
Subramani, Arun
Szilagyi, Istvan
Toshinori, Tsuru
Tseng, Hui-hsin
Vankelecom, Ivo F.J.
Watanabe, Toshio
Wechsler, Daniel S.
Whittaker, Jonathan
Wolf, Julie
Wong, Chung
Wu, Bing
Wu, Cuiming
Yu, Jianjia
Yuan, Hongyan
Zhao, Shuaifei

